# Graphene Oxide-Coated Patterned Silk Fibroin Films Promote Cell Adhesion and Induce Cardiomyogenic Differentiation of Human Mesenchymal Stem Cells

**DOI:** 10.3390/biom13060990

**Published:** 2023-06-14

**Authors:** Jie Wang, Yi Wu, Yecheng Wang, Yajun Shuai, Zongpu Xu, Quan Wan, Yuyin Chen, Mingying Yang

**Affiliations:** Key Laboratory of Silkworm and Bee Resource Utilization and Innovation of Zhejiang Province, Institute of Applied Bioresource Research, College of Animal Science, Zhejiang University, 866 Yuhangtang Road, Hangzhou 310058, China; wangjie1987@zju.edu.cn (J.W.);

**Keywords:** silk fibroin, graphene oxide, mesenchymal stem cells, cardiomyogenic differentiation, myocardial repair

## Abstract

Cardiac tissue engineering is a promising strategy for the treatment of myocardial damage. Mesenchymal stem cells (MSCs) are extensively used in tissue engineering. However, transformation of MSCs into cardiac myocytes is still a challenge. Furthermore, weak adhesion of MSCs to substrates often results in poor cell viability. Here, we designed a composite matrix based on silk fibroin (SF) and graphene oxide (GO) for improving the cell adhesion and directing the differentiation of MSCs into cardiac myocytes. Specifically, patterned SF films were first produced by soft lithographic. After being treated by air plasma, GO nanosheets could be successfully coated on the patterned SF films to construct the desired matrix (P-GSF). The resultant P-GSF films presented a nano-topographic surface characterized by linear grooves interlaced with GO ridges. The P-GSF films exhibited high protein absorption and suitable mechanical strength. Furthermore, the P-GSF films accelerated the early cell adhesion and directed the growth orientation of MSCs. RT-PCR results and immunofluorescence imaging demonstrated that the P-GSF films significantly improved the cardiomyogenic differentiation of MSCs. This work indicates that patterned SF films coated with GO are promising matrix in the field of myocardial repair tissue engineering.

## 1. Introduction

Mesenchymal stem cells (MSCs) implantation is a promising strategy for myocardial repair because MSCs are able to attenuate cardiac fibrosis and promote neovascularization by secreting reparative paracrine factors [1,2,3]. More importantly, MSCs are pluripotent cells with the ability to differentiate into cardiac myocytes, which replenish damaged heart cells, since cardiac myocytes are incapable of regenerating themselves after birth [4,5,6]. Although there is great potential for cardiac repair, the therapeutic efficiency of MSCs implantation is still limited due to poor adhesion [7,8] and low efficacy of conversion into cardiac myocytes [9,10]. Designing artificial matrices to support cell growth has been proposed as an effective method for improving cell adhesion and proliferation, and even for directing pluripotent cell differentiation in tissue engineering. Artificial matrices consisting of collagen protein and featuring nanofibrous structure, which mimic the composition and structure of the extracellular matrix (ECM), have been reported to play an important role in modulating stem cells’ fate [11,12,13,14]. Thus, a structured biological-derived matrix is expected to enhance the adhesion and mediate the differentiation of MSCs for cardiac tissue engineering. 

Silk fibroin (SF) derived from *Bombyx mori* (*B. mori*) silkworm is an ideal biomaterial in tissue engineering. It has unique qualities including biocompatibility, plasticity, biodegradability, and excellent mechanical properties [15,16], enabling SF to serve as a biomimetic platform for supporting cell growth [17,18,19]. In addition, silk fibroin matrix with an elaborated topological structure can be easily fabricated by lithography or electrospinning techniques [20,21,22]. Some reports have demonstrated that patterned SF films are able to guide the osteogenic or neurogenic differentiation of MSCs [23,24,25]. Meanwhile, cardiac muscle tissue is characterized by anisotropy, contributing to its powerful contraction properties [26] and specific cellular orientation and elongation [14]. Therefore, topologically structured SF matrices hold great promise for cardiac tissue engineering. Nonetheless, how to improve the adhesive properties and biological function of the matrix are urgent issues that remain to be solved, because SF provides only a biophysical inducing factor for differentiation and generally low adhesion to cells. 

Meanwhile, graphene oxide (GO), an oxidized derivative of graphene sheets, has received much attention in biomedical application [27,28]. It contains active oxygen groups, providing numerous binding sites to absorb proteins, carbohydrates, and stimulatory factors [29,30,31,32], which are essential components in mediating the adhesion and directional differentiation of MSCs. Moreover, with high hydrophilicity and colloidal stability, GO has been widely used in surface functionalization modification and composite materials construction [33,34,35]. Attempts have been made to produce GO and SF (GO/SF) composites, such as nanocomposite membranes, scaffolds, and nanofibers [36,37,38]. The present GO/SF composites exhibit improved mechanical and electrical ability, but less emphasis has been placed on their biological properties. Meanwhile, functional surface coatings with GO provide an alternative strategy for the enhancement of the bioactivity of substrates [39,40]. GO-based coatings are extremely stable and require no additional chemical treatment during the process. Therefore, we expected that GO and SF composite matrix (GSF), prepared by coating GO onto a patterned SF film, could form a nano-topological surface structure and display functional groups of GO, thereby triggering the cellular adhesion and cardiomyogenic differentiation of MSCs. 

**Figure 1:**
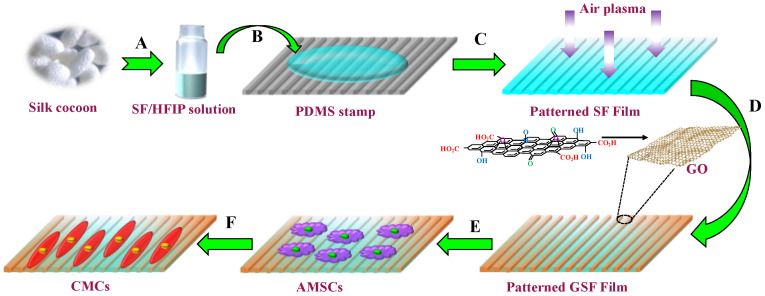
Schematic diagram depicting the formation of P-GSF patterned films by the soft lithographic method and coated with GO nanosheets as a matrix for myocardial differentiation of AMSCs. (**A**) SF working solution was first prepared by dissolving regenerated SF with HFIP; (**B**) SF working solution was dropped onto the PDMS stamp; (**C**) pattern SF films were generated after air-drying followed by air plasma treatment; (**D**) monodispersed graphene oxide solution was coated onto P-SF films. (**E**) P-GSF films were formed following the solvent evaporation and peeling off of film, and human AMSCs were cultured on the P-GSF films; (**F**) AMSCs were induced to differentiate into cardiac myocytes (CMCs) under the function of P-GSF matrix.

To develop such a matrix, we first designed patterned SF films by using a soft lithographic technique. An SF working solution was prepared by dissolving SF in an organic solvent (Figure 1A). The patterned SF films were fabricated by casting the working solution onto PDMS stamps with a linear groove structure, followed by natural drying (Figure 1B). The surfaces of the patterned SF films were then treated with air plasma to activate the functional groups of SF (Figure 1C). Following that, we introduced GO nanosheets for coating the surface of the patterned SF films to produce the patterned GSF (P-GSF) matrix (Figure 1D). We used human adipose-derived mesenchymal stem cells (AMSCs) to prove that the P-GSF films presented improved performance in cell adhesion and an orientated tendency in cell growth, and further directed cellular differentiation into cardiac myocytes (Figure 1E,F).

## 2. Materials and Methods

### 2.1. Fabrication of P-GSF Films

Lyophilized SF powder was derived from *B. mori* cocoons by following our previous protocol [41]. The powder was dissolved in hexafluoroisopropanol (HFIP) to acquire a working solution with concentration of 6 wt%. Poly(dimethylsiloxane) (PDMS, Sylgard 184, Dow Corning, Midland, TX, USA) stamp was used in our study to produce the patterned surface of the matrix. PDMS prepolymer was first cast on a silicon substrate with linear groove topography surface (period: 606 nm, width: 330 nm, depth: 190 nm, GermanTech Co., Ltd., Beijing, China). Then, the PDMS stamp was peeled from the silicon substrate after solidification for 2 h at 85 °C. The PDMS stamp was cleaned with acetone and isopropanol, respectively, several times before use. Patterned SF films (P-SF) were fabricated by casting 100 µL of SF working solution on the PDMS stamp and air-drying at room temperature. The films were soaked in 80% methanol solution for 2 h to induce insolubility in water. Then, the P-SF films were treated with air plasma for 1 min at a 50 W radio frequency. For coating GO nanosheets onto the surface of P-SF films, GO aqueous solution with various contents (0.05–1%) was added dropwise onto the films. After natural drying, the films were washed with deionized water and vacuum dried at room temperature.

### 2.2. Characterization of P-GSF Films

The protein adsorption of P-GSF films with various GO contents was evaluated using bovine serum albumin (BSA) and fetal bovine serum protein (FBS), respectively, following the protocol of the BCA protein assay kit (Beyotime Biotechnology, Beijing, China). The mechanical properties of the films were determined by the tensile tests, in which the films were cut into strip shapes and the stretch speed was 0.1 mm/s. The respective surface topographies of GO-coated SF films (GSF), patterned SF films (P-SF), and GO-coated patterned SF films (P-GSF) were obtained by scanning electron microscope (SEM). Raman spectra and Raman scanning images of films were analyzed under a 532 nm laser irradiation by microscopic imaging Raman spectrometer (DXR2, Thermo Fisher Scientific, Waltham, MA, USA). The surface hydrophilicity of films was characterized by measurement of the water contact angle.

### 2.3. Cell Adhesion Assay

Human adipose-derived mesenchymal stem cells (hAMSCs, Cyagen Biosciences, Guangzhou, China) were used in our study and cultured in a complete medium containing FBS, penicillin–streptomycin, and L-glutamine (HUXMD-90011, Cyagen Biosciences, Guangzhou, China). The GSF, P-SF, and P-GSF films were pretreated with 75% ethanol for 2 h for sterilization and were rinsed with PBS three times. The films were then placed on the bottom of 24-well plates, and a tissue culture plate (TCP) was set as the control group. hAMSCs at a density of 1.0 × 10^4^ cells/cm^2^ were seeded on different films and the control plates. After culturing for 1 h and 4 h, respectively, the cells in all groups were washed three times with PBS to remove the non-adherent cells. Following that, the cells were fixed in 4% formalin solution for 30 min and permeated in 1% Triton X-100 for 10 min. The cytoskeleton was stained with 480 phalloidin dye for 30 min and observed under a confocal microscopy system (LSM710, Carl Zeiss, Oberkochen, Germany) to analysis the cell adhesion. Immunofluorescence staining of cell adhesion proteins (vinculin and paxillin) was also carried out to study further the cell adhesion properties. hAMSCs were cultured for 24 h and treated with 4% formalin and 1% Triton X-100 in turn. The cells were subsequently blocked with 5% BSA for 30 min at room temperature. After washing with PBS three times, the hAMSCs were incubated with primary antibodies (anti-vinculin from Invitrogen, anti-paxillin from Abcame, Cambridge, UK) overnight at 4 °C, followed by treatment with Alexa Fluor 488 (Abcame, Cambridge, UK) and Alexa Fluor 594 (Abcame, Cambridge, UK), respectively, for 30 min. Finally, after washing with PBS three times, the fluorescence was observed with LSCM. The semiquantitative analysis of the mean fluorescence intensity of the adhesion protein was performed in Image J software (V 1.8.0).

### 2.4. Cell Morphology and Viability Assay

hAMSCs at a density of 1.0 × 10^4^ cells/cm^2^ were seeded on 24-well plates, covered with different films and cell plate, respectively. After being cultured for 24 h, the cells’ cytoskeletons were stained by 480 phalloidin dye according to the above-mentioned protocol. The fluorescence images of cell morphology were acquired under LSCM. The morphologies of hAMSCs in different groups were also characterized using scanning electron microscopy (SEM, SU8010, Hitachi, Tokyo, Japan). The samples were first fixed with 2% glutaraldehyde overnight at 4 °C and then washed with PBS twice. After that, cells were treated with 1% osmic acid for 1 h and dehydrated with gradient ethanol for 15 min each time. Finally, the samples were dried with a critical point drier and coated with gold before SEM observation. Pseudo-color processing was used in the image analysis to highlight the cells’ morphology.

The cell viability of hAMSCs on different films were assessed using a Cell Counting Kit-8 (CCK-8, Dojindo, Tokyo, Japan). After hAMSCs were cultured for 1 d, 3 d, and 5 d on the 96-well plates covered with films, 10 µL of working solution was added and incubated for another 2 h at 37 °C. After that, 100 µL of supernatant from each well was transferred to the new 96-well plates, and the absorbance of each well was determined at 450 nm. 

### 2.5. Cardiomyogenic Differentiation of hAMSCs on P-GSF Films

For cardiomyogenic differentiation, the hAMSCs (1.0 × 10^4^ cells/cm^2^) at passage 4–5 were implanted on 6-well plates covered with different films and cultured in the complete medium (Cyagen Biosciences, Guangzhou, China) for 24 h. Then, the medium was discarded and the cells were treated with an inducing medium (complete medium containing 10 µM 5-azacytidine (5-aza)) for 24 h. After that, the inducing medium was replaced with new complete medium and cultured for 14 d and 28 d, respectively. The culture medium was changed every three days. 

Cardiac troponin T (cTnT) and connexin 43, two important effector proteins of cardiomyocytes, were selected and detected by immunofluorescence staining. The hAMSCs were fixed with 4% formalin and blocked in 2% BSA. Then, the cells were incubated with the primary antibodies (anti-cTnT and anti-connexin 43) overnight at 4 °C, followed by staining with the secondary antibody (Alexa Fluor 594, Abcame Cambridge, UK) at room temperature for 1 h. Finally, the cells were stained by 480 phalloidin to indicate the cytoskeleton. For real-time polymerase chain reaction (PCR), the total RNA of hAMSCs cultured on different films was extracted using a total RNA extraction kit (Solarbio, Beijing, China). After reverse transcription and purification, the mRNA levels of cTNT, connexin 43, and GATA binding protein 4 (GATA 4) of hAMSCs in different groups were determined by real-time RT-PCR analysis using a TaqMan primer-probe. All primers (Sangon Biotech, Shanghai, China) were designed and are listed in Table 1 with GAPDH as a reference gene.

### 2.6. Statistical Analysis

The data were calculated and are presented as mean values ± standard deviation (SD), *n* = 3. One-way analysis of variance was adopted to statistically analyze the data. Differences between groups were considered statistically significant at *p <* 0.05, and extremely significant at *p ≤* 0.01.

## 3. Results

### 3.1. Formation and Characterization of P-GSF Films

**Figure 2:**
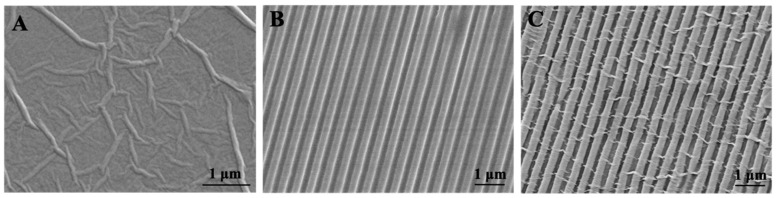
Surface morphology of (**A**) GSF, (**B**) P-SF, and (**C**) P-GSF films visualized by SEM.

The surface micro-morphology of patterned SF films was observed by SEM (Figure 2). As expected, the P-SF films featured dimensions including a linear groove structure with a line width of about 300 nm and depth of nearly 200 nm (Figure 2B), largely replicated from the PDMS stamps. As a control, the GSF films exhibited the typical ridge structure of GO (Figure 2A). The P-GSF films turned from transparent to deep brown as the content of GO coating increased (Figure S1A). Protein adsorption on the films was analyzed to evaluate their biocompatibility. The results indicated that the GO coating promoted protein adsorption (Figure S1B,C); P-GSF films with a higher GO content can adsorb more BSA or FBS in the first 24 h, implying that it can enhance cell attachment. GO coating also resulted in an increase in the elastic modulus of P-GSF films (Figure S1D). When the content of GO increased to 0.25%, the elastic modulus of P-GSF films increased to 93 MPa, twice that of P-SF films. However, when the content of GO was higher than 1%, the P-GSF films became fragile. This is because the high content of GO in P-GSF films may lead to an increase of rigidity and a decrease of toughness. SEM indicated GO with a nanofiber morphology evenly distributed on the groove or ridge surface of the P-SF films (Figure S2). Compared with P-GSF films with GO content at 0.25%, P-GSF films of higher GO content (0.5% and 1%), showed an increase in the size of the GO nanofibers and a decrease in the width of the grooves, finally completely covering the patterned surface of the P-SF films. This means that the GO coating with a content of 0.25% was more suitable for constructing a P-GSF matrix with a desirable surface structure (Figure 2C) for directing the orientation and promoting the adhesion of AMSCs. Raman spectroscopy presented peaks at 1600 cm^−1^ and 1340 cm^−1^, which can be assigned to the G band and D band of GO, further confirming the coating with GO (Figure S3A). Raman imaging indicated that GO was evenly distributed on the surface of the P-SF films (Figure S3B). The water contact angle of the SF and P-SF films was 76.5° and 69.4°, respectively. However, the water contact angle of GSF and P-GSF films decreased to 60.4° and 58.0°, respectively, implying that GO coating treatment improved the hydrophilicity of the SF films because of the introduction of oxygen-compound groups from GO (Figure S3C). 

### 3.2. Cell Adhesion of AMSCs

**Figure 3:**
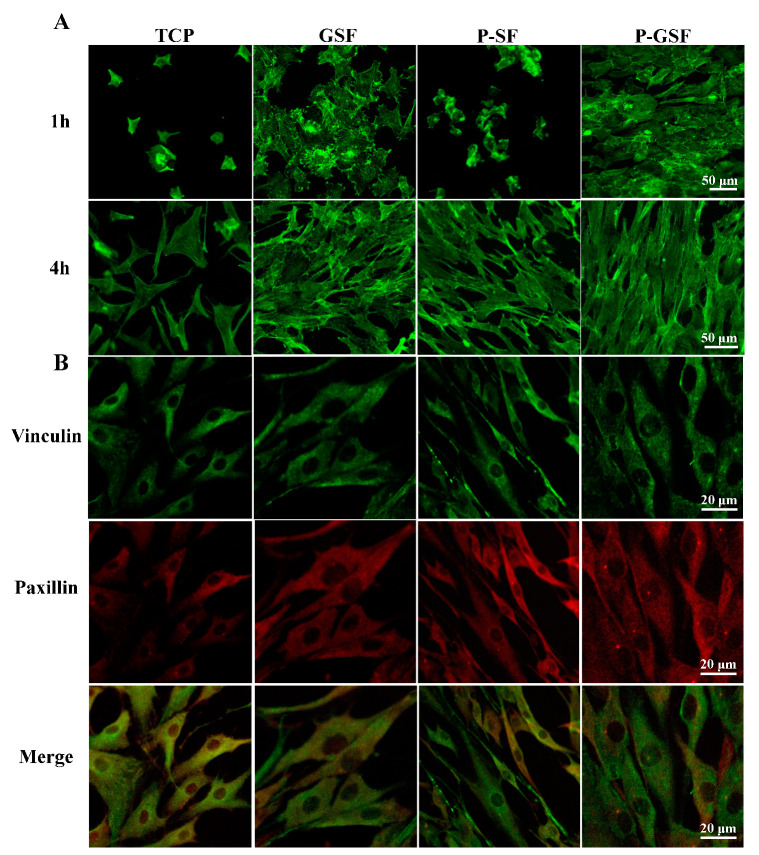
Cell adhesion morphology and adhesion-associated protein analysis of AMSCs on TCP, GSF films, P-SF films, and P-GSF films. (**A**) The adhesion morphology of AMSCs in different groups cultured for 1 and 4 h. (**B**) Adhesion-associated proteins of cells cultured on TCP, GSF films, P-SF films, and P-GSF films, visualized by immunofluorescence staining (**B**).

We used human adipose mesenchymal stem cells (hAMSCs) to investigate the early cell adhesion to the GSF, P-SF, and P-GSF films (Figure 3A). Cells cultured on TCP plates were used as a control. Relatively few HAMSCs were observed in the TCP and P-SF groups after culturing for 1 h, whereas more cells adhered on the GSF and P-GSF films and exhibited a more stellate-patterned phenotype. After culturing for 4 h, rapid hAMSCs spreading was observed on the GSF and P-GSF films, but those cultured on the TCP plates remained spindled or narrow, a morphological feature of non-spreading hAMSCs. The results indicated that GO coating successfully enhanced cell adhesion on the SF matrix and promoted cell spreading on the surface of the GSF and P-GSF films. In addition, two adhesion-associated proteins of hAMSCs, vinculin and paxillin, were also labelled by immunofluorescence staining. As shown in Figure 3B, in all groups, vinculin and paxillin were obviously expressed around the cell nucleus, indicating that the two proteins played a role in the adhesion of hAMSCs. The fluorescence intensity of paxillin in hAMSCs cultured on the P-GSF films was higher than for the other three groups (Figure S4), indicating that the GO coating and patterned structure both contributed to the cell adhesion. 

### 3.3. Cell Proliferation and Cell Morphology of AMSCs

**Figure 4:**
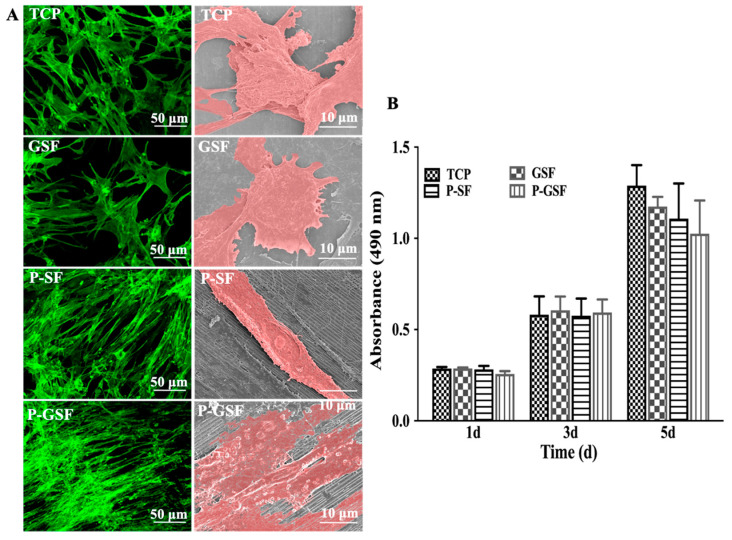
Growth morphology and proliferation of AMSCs on different films. (**A**) Growth morphology of AMSCs on TCP, GSF, P-SF, and P-GSF films cultured for 24 h. Left column shows fluorescence images by cytoskeleton staining, right column shows the relative scanning electron microscope images with pseudo-color treatment. (**B**) Cell proliferation of AMSCs in different groups cultured for 1, 3 and 5 d, determined by MTS analysis.

The cell morphology of AMSCs cultured on TCP, GSF, P-SF and P-GSF for 24 h was characterized by CLSM and SEM, respectively (Figure 4A). AMSCs exhibited a starfish-shaped morphology when grown on TCP and GSF films, without obvious orientation distribution. By contrast, AMSCs on the patterned surfaces of P-SF and P-GSF films were oriented along the direction of grooves, with a spindle-shaped phenotype morphology. The results demonstrated that the designed pattern provided suitable guidance to lead the cell alignment. The cell proliferation of AMSCs was analyzed by culturing AMSCs on different matrices for 1, 3, and 5 d, respectively. Figure 4B shows that the absorbance of all groups increased over time, indicating the positive proliferation of AMSCs cultured on the substrate throughout the whole culture period. Cells cultured for 5 d were lower in number on the P-GSF films than the other three matrices, without statistical difference. This may be because some AMSCs cultured on P-GSF films progressed to differentiation rather than continually proliferating. Therefore, the results indicate that P-GSF films could serve as a matrix to maintain cell growth.

### 3.4. Cardiomyocytes Differentiation and Cardiac-Specific Gene Expression of AMSCs

**Figure 5:**
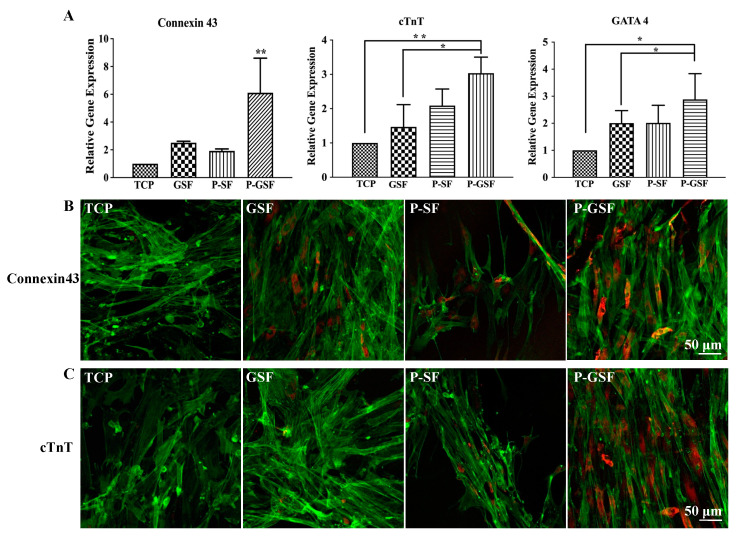
Assessment of cardiomyogenic differentiation of AMSCs on TCP, GSF, P-SF, and P-GSF films. (**A**) Relative levels of mRNAs for connexin 43, cTnT, and GATA4 of AMSCs cultured on different films for 28 d. Immunofluorescence images of (**B**) connexin 43 and (**C**) cTnT proteins in human AMSCs seeded in different groups for 28 d. Connexin 43 and cTnT were stained by Alexa Fluor 594-labeled antibody (red). Cell cytoskeletons were stained with phalloidin (green). * *p* < 0.05, ** *p* < 0.01, data are presented as mean ± SD, *n* = 3.

We investigated whether the culture of AMSCs on P-GSF films promoted their cardiomyogenic differentiation by evaluating myocyte-related gene expression through a qRT-PCR assay. The gene expression of gap junction proteins (connexin 43) and cardiomyogenic contractile proteins (cTnT) were significantly higher in the P-GSF group compared with TCP, GSF, and P-GSF groups on day 14 and day 28 (Figure S5A and Figure 5A). Cardiomyogenic transcriptional factor (GATA4) expression exhibited no difference among all groups when AMSCs were cultured in the earlier period (day 14), and its expression was maximal in the P-GSF group on day 28. This means that transformation of AMSCs into cardiomyocytes may have been activated in the early days and finally completed in the later period. The gene expression of connexin 43 was higher in the GSF group than the TCP or P-SF groups, while cells cultured on P-SF films expressed more cTnT than TCP or GSF. This is because the GO coating focused on the promotion of cells adhesion and junction, but the patterned structure provided an inducer of mechanical stress for cells. In addition, immunofluorescence staining of connexin 43 and cTnT confirmed the gene analysis results (Figure 5B and Figure S5B). When cultured on day 14, AMSCs cultured on the P-GSF films had recognizable connexin 43-positive and cTnT-positive staining, but no significant red fluorescence was observed in the other groups (Figure S5B). On day 28, red fluorescence was observed in the GSF, P-SF, and P-GSF groups, and the P-GSF group had the most positive cells expressing connexin 43 or cTnT protein. Taken together, the above results consistently verify that the P-GSF films can act as an ideal matrix to direct the cardiomyogenic differentiation of AMSCs. In our study, 5-aza was added into the medium to induce cardiomyogenic differentiation in first 24 h, according to previous studies. Therefore, we analyzed the retention ability of 5-aza on different matrices. As expected, P-GSF films can retain more 5-aza than GSF, P-SF, and TCP after 24 h (Figure S6), indicating that more inducer may participate in promoting the differentiation of cardiac myocytes.

## 4. Discussion

Cardiomyocyte differentiation of stem cells has always been a great challenge. Cardiomyocytes are sensitive to a series of growth factors, including connexin 43, endothelial growth factor (VEGF), and fibroblast growth factor-2 (FGF-2) [42,43,44], and it takes a long time to complete the transformation of pluripotent cells into cardiomyocytes. In this study, we constructed an artificial matrix consisting of SF and GO to explore the possibility of cardiomyocyte differentiation by stem cells on our matrix to facilitate myocardial repair by tissue engineering. 

Numerous studies have shown that cell morphology, viability, and even biological function can be affected by the surrounding environment of cells [45,46]. Mature cardiomyocytes exhibit a fibrous structure. Therefore, we firstly designed a patterned SF film (Figure 2), providing conditions that included a topographic structure to direct the growth of stem cells (Figure 4). Meanwhile, graphene oxide (GO) is regarded as an excellent functional material. The sheet structure and large number of bonding sites enable GO to efficiently bind or stimulate biological factors when used in vivo. Byung-Soo Kim considered that GO can potentiate the myocardial repair efficacy of mesenchymal stem cells by stimulating the expression of angiogenic growth factors and gap junction proteins [47]. However, the scaffolds from GO alone are fragile, although GO is reported to be of high mechanical strength. Our results also proved that excess GO (higher than 1%) attenuated the elastic modulus of composite GO-SF films (Figure S1D). Furthermore, the biosafety of GO is another concern. In our study, a coating method was applied to introduce GO into our matrix. The proliferation results for the cells indicated that the P-GSF films were biocompatible (Figure 4). We also conducted an adsorption experiment with proteins (FBS and BSA) and chemical substances (5-aza). FBS is an essential nutrient component in the culture of cells, while 5-aza plays an important role in inducing the cardiomyocyte differentiation of stem cells. The results indicated that the adsorption of FBS and 5-aza significantly increased when GO was coated on the matrix (Figures S1C and S6). This may explain why stem cells cultured on P-GSF films had faster adhesion behavior and higher efficiency of cardiomyocyte differentiation. 

Overall, our results for cell morphology, adhesion, and differentiation confirmed the underlying mechanisms which make P-GSF films suitable for myocardial repair tissue engineering. In the follow-up study, we will conduct in vivo research to further validate the efficiency of our matrix for the repair of myocardial injury.

## 5. Conclusions

We designed a novel matrix using SF films coated with GO nanosheets to promote the cell adhesion and cardiomyogenic differentiation of MSCs. The resultant P-GSF films have a nano-topographic surface with ordered grooves interlaced by GO ridges. In the testing, the P-GSF films exhibited a higher hydrophilicity and protein adsorption capacity than P-SF films, which further promoted the early adhesion of MSCs. More importantly, the P-GSF films directed the growth orientation of MSCs into a linear arrangement, and induced their differentiation into cardiac myocytes, as demonstrated by relative gene expression and immunofluorescence. Our work indicates that P-GSF films are promising matrices for supporting the growth and differentiation of MSCs into cardiac myocytes for enhancing myocardial tissue regeneration. 

## Figures and Tables

**Table 1:** Primer Sequences Used for Reverse Transcription Polymerase Chain Reaction Gene Expression Analysis.

Genes	5′-3′	Primers
*GAPDH*	forward	TGACGCTGGGGCTGGCATTG
	reverse	GGCTGGTGGTCCAGGGGTCT
*cTnT*	forward	GGCAGCGGAAGAGGATGCTGAA
	reverse	GAGGCACCAAGTTGGGCATGAACGAC
*Connexin 43*	forward	ACT GGC GAC AGA AAC AAT TCT TC
	reverse	TTC TGC ACT GTA ATT AGC CCA GTT
*GATA-4*	forward	TCCCTCTTCCCTCCTCAAAT
	reverse	TCAGCGTGTAAAGGCATCTG

## Data Availability

Not applicable.

## Supplementary Materials

The following supporting information can be downloaded at: https://www.mdpi.com/article/10.3390/biom13060990/s1, Figure S1: Characterization of P-GSF films; Figure S2: Surface morphology of P-GSF films in different contents of GO coating visualized by SEM; Figure S3: The Raman spectra analysis and surface wettability evaluation of different films; Figure S4: The mean fluorescence intensity of vinculin and paxillin proteins treated by immunofluorescence staining; Figure S5: Assessment of cardiomyogenic differentiation of AMSCs on TCP, GSF, P-SF, and P-GSF films; Figure S6: Adsorption and retention comparison of 5-AZA of TCP, P-SF, GSF, and P-GSF films.
