# Supplementary material for: Graphene Oxide-Coated Patterned Silk Fibroin Films Promote Cell Adhesion and Induce Cardiomyogenic Differentiation of Human Mesenchymal Stem Cells
Source: Biomolecules. 2023 Jun 14;13(6):990. doi: 10.3390/biom13060990 (PMC10296049; doi:10.3390/biom13060990)
Supplement: Supplementary file 1 [file biomolecules-13-00990-s001.zip › biomolecules-2420809-supplementary.pdf]

Supporting Information

Graphene Oxide-Coated Patterned Silk Fibroin Films Promote Cell Adhesion and Induce Cardiomyogenic Differentiation of Human Mesenchymal Stem Cells

Jie Wang, [a] *Yi Wu,* [a] *Yecheng Wang,* [a] *Yajun Shuai,* [a] *Zongpu Xu,* [a] *Quan Wan,* [a] *Yuyin Chen* [a] *and Mingying Yang* *[a]

a. Key Laboratory of Silkworm and Bee Resource Utilization and Innovation of Zhejiang Province, Institute of Applied Bioresource Research, College of Animal Science, Zhejiang University, Yuhangtang Road 866, Hangzhou 310058, China.

Email: yangm@zju.edu.cn

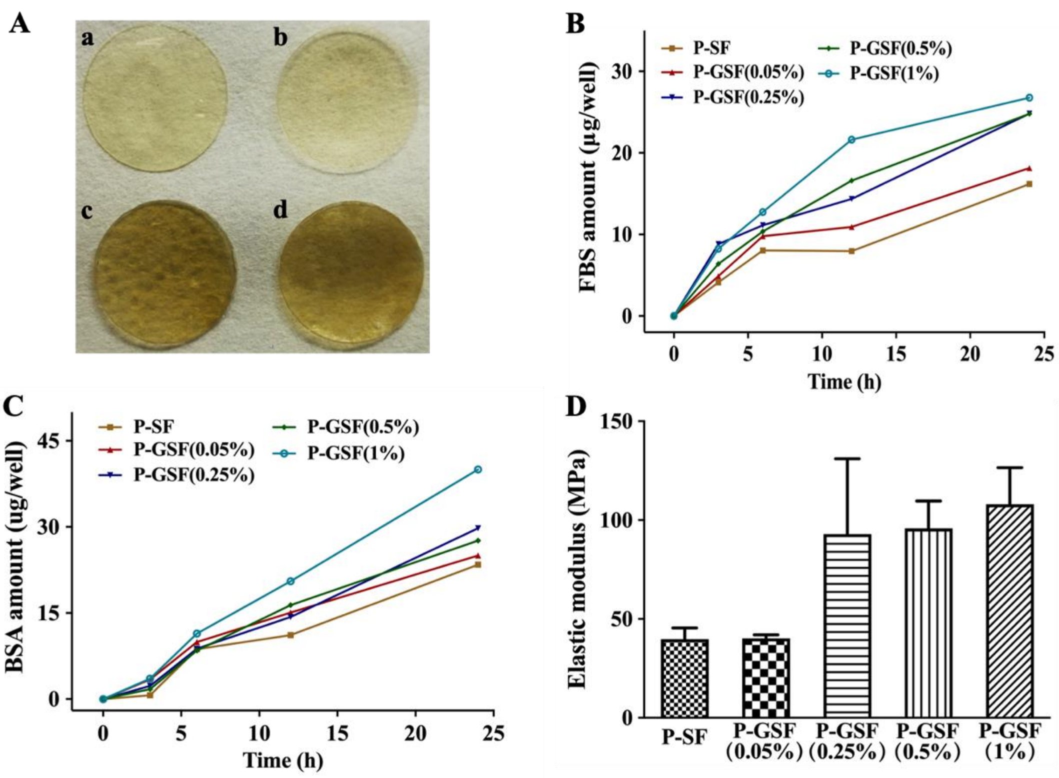

Figure S1. Characterization of P-GSF films. (A) Photograph of P-GSF films with contents of GO coating, a-0.05%, b-0.25%, c-0.5%, d-1%. FBS (B) and BSA (C) proteins adsorption analysis of P-GSF films in various GO contents. (D) Elastic modulus of P-GSF films with various contents of GO coating.

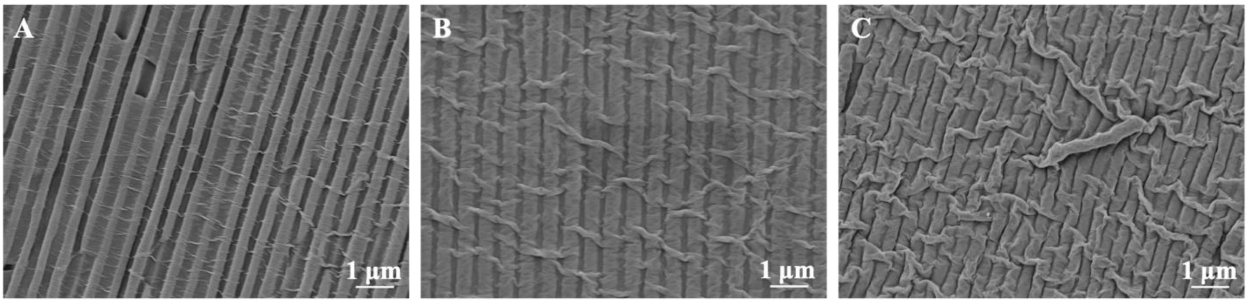

Figure S2. Surface morphology of P-GSF films in different contents of GO coating visualized by SEM. The contents of GO is 0.25 % (A), 0.5 % (B) and 1 % (C), respectively.

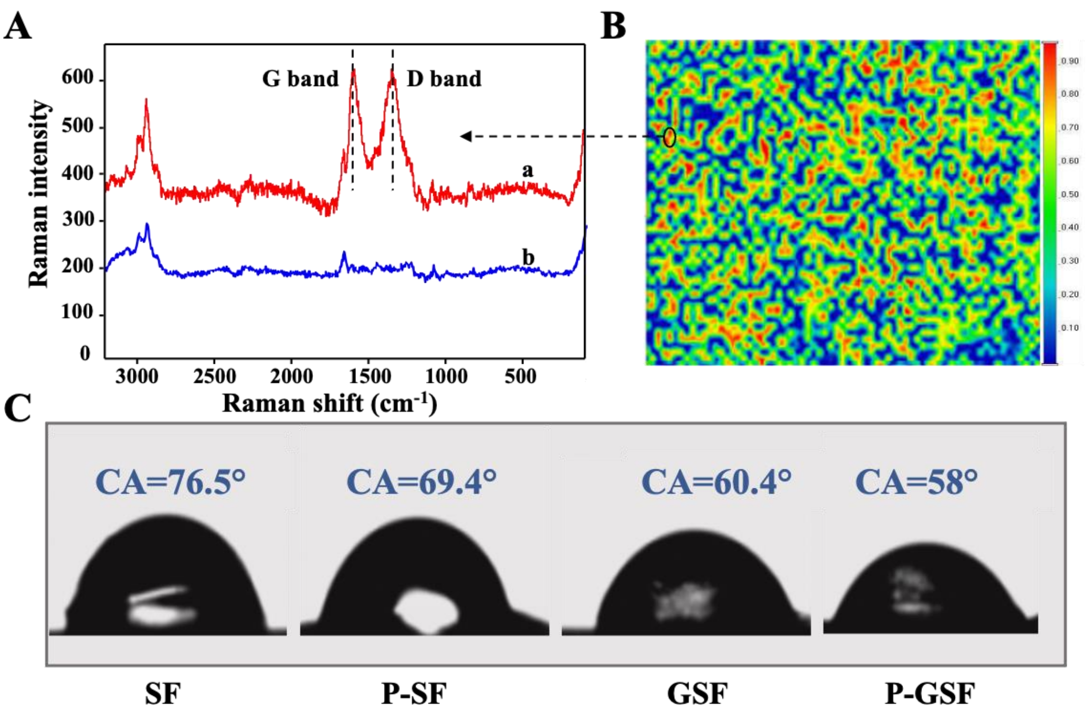

Figure S3. The Raman spectra analysis and surface wettability evaluation of different films. (A) The Raman spectra of P-GSF film (a) and P-SF film (b). (B) A representative Raman scanning image of randomly selected area P-GSF film. High signal intensity (red) means the existence of GO. (C) The surface wettability of SF, P-SF, GSF and P-GSF films was evaluated by water contact angle measurement.

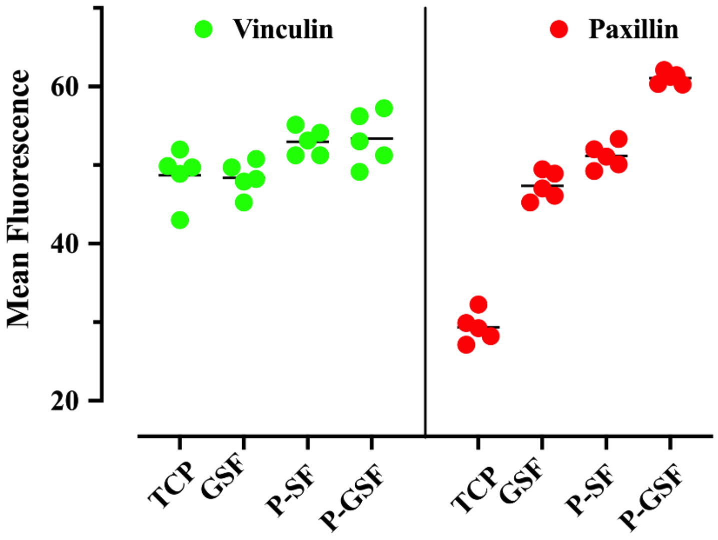

Figure S4. The mean fluorescence intensity of vinculin and paxillin proteins treated

by immunofluorescence staining. The semiquantitative analysis of mean fluorescence intensity was carried in Image J software.

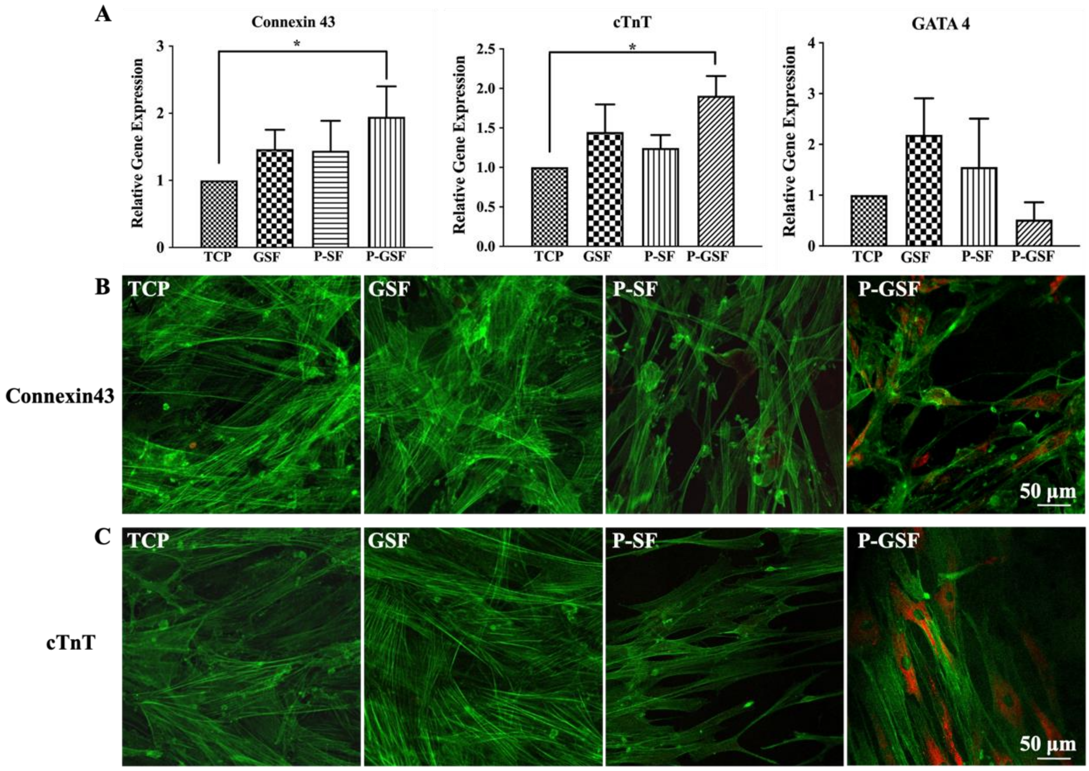

Figure S5. Assessment of cardiomyogenic differentiation of AMSCs on TCP, GSF, P-SF and P-GSF films. (A) Relative levels of mRNAs for Connexin 43, cTnT and GATA4 of AMSCs for 14 d cultured in different films. Immunofluorescence images of Connexin 43 (B) and cTnT (C) proteins in human AMSCs seeded for 14 d in different groups. Connexin 43 and cTnT were stained by Alexa Fluor 594-labeled antibody (red). Cell cytoskeleton were stained with phalloidin (green). $^{*}p < 0.05$, data are presented as mean ± SD, $n = 3$.

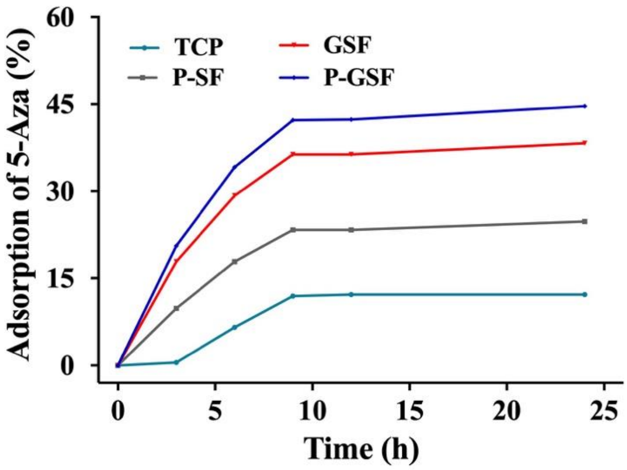

Figure S6. Adsorption and retention comparison of 5-AZA of TCP, P-SF, GSF and P-GSF films.
